# Can the interleukin TNF-α be used as a biomarker for pulp necrosis?

**DOI:** 10.2340/aos.v84.43034

**Published:** 2025-02-27

**Authors:** Carlos Wesley Lopes Brasil da Silva, Beatriz Melare de Oliveira, Jheinis Stefany Pascuineli Duarte, Thalya Fernanda Hortsh Maltarollo, Henrico Badaoui Strazzi-Sahyon, Gustavo Sivieri-Araujo

**Affiliations:** a Department of Preventive and Restorative Dentistry, Discipline of Endodontics, Araçatuba School of Dentistry, São Paulo State University – UNESP, Araçatuba, Brazil; b Department of Prosthodontics and Periodontology, Bauru School of Dentistry, University of São Paulo – USP, Bauru, Brazil; c Division of Biomaterial and Biomedical Sciences, Department of Oral Rehabilitation and Biosciences, Oregon Health & Science University, OHSU, Portland, OR, USA

**Keywords:** Tumor necrosis factor-alpha, biomarkers, dental pulp necrosis

Dear Editor,

Apical periodontitis and pulp necrosis are conditions that arise from the host’s immune response to microbial invasion within the dental pulp. This inflammatory process progressively degrades pulp tissue and, in advanced stages, may lead to necrosis. These diseases are clinically significant and highly prevalent worldwide, with recent studies showing that apical periodontitis affects roughly 50% of the global adult population. The prevalence is even higher in dental clinic populations and hospital settings [1].

A crucial component of the inflammatory response is the release of cytokines and interleukins, particularly interleukin-1β and tumor necrosis factor-α (TNF-α), both of which play significant roles in tissue breakdown during pulpitis [2]. Notably, TNF-α has been observed to increase protein levels during pulp inflammation, potentially correlating with the progression of inflammatory damage [2].

Patients with comorbidities such as type 2 diabetes face unique challenges due to the interplay between systemic conditions and local inflammatory responses, which can complicate diagnosis and idealize treatment strategies. Consequently, the application of biomarkers holds significant potential for facilitating earlier diagnoses and guiding optimal therapeutic approaches in such cases. Biomarkers, defined as measurable indicators of a biological state or condition, can provide valuable insights into the degree of tissue inflammation, thereby enhancing diagnostic precision and informing targeted therapeutic interventions [3].

Accordingly, biomarkers capable of indicating the degree of pulp inflammation and the onset of necrosis hold significant clinical value by enabling more conservative treatment approaches and improving the strategic planning of clinical interventions.

## Definition of biomarker

A biomarker is a biological observation that predicts a clinically relevant outcome, as defined by the NIH Definitions Working Group (2000) and the Biomarkers Definitions Working Group (2001) [3]. Biomarkers are widely utilized and serve as essential tools in various diagnostic contexts.

In a more recent definition, a biomarker can be described as ‘a functional variant or quantitative index of a biological process that predicts or reflects the evolution or predisposition to a disease or a response to therapy’ [3]. This definition highlights the critical role of biomarkers not only in diagnosing conditions but also in monitoring disease progression and therapeutic responses.

Thus, the primary objective of a biomarker is to facilitate precise clinical diagnoses of patient conditions, ensuring replicability and accuracy. These characteristics are essential for a biomarker to be considered effective and reliable in clinical practice.

## Involvement of TNF-α in pulp necrosis

Interleukins are closely linked to inflammatory processes, and in the context of pulp necrosis and the underlying mechanisms of this pathological condition, TNF-α plays a pivotal role in bone resorption within periapical lesions [4, 5]. TNF-α is critical in modulating the inflammatory response in pulp tissues, being integral to pathological processes such as pulp necrosis, as detailed by Taira et al. [4]. Upon activation by inflammatory stimuli, TNF-α binds to specific receptors on target cells, initiating a signaling cascade that results in the expression of pro-inflammatory cytokines and the activation of osteoclasts, which are responsible for bone resorption [6]. Additionally, TNF-α activation, via the ERK (extracellular signal-regulated kinase) pathway, not only promotes the differentiation of odontogenic cells but also enhances osteoclastic activity [4, 6].

The presence of comorbidities such as type 2 diabetes can complicate the inflammatory response in the dental pulp and influence the progression of pulp necrosis. Recent studies, such as that by Agrawal et al. [5], support the hypothesis that TNF-α concentration in diabetic patients with pulpal inflammation is significantly altered, reflecting the interaction between systemic conditions and local inflammatory processes. However, Alsamahi et al. [7] emphasize that the expression of inflammatory cells in diabetic patients is associated with macrophage markers, indicating an exacerbated immune response. This supports the use of biomarkers to develop more effective therapeutic strategies.

Furthermore, TNF-α exhibits a synergistic response in the context of an induced inflammatory environment, with an increase in osteoclasts corresponding to elevated interleukin concentrations, as demonstrated by Taira et al. [4]. Additionally, in the context of type 2 diabetes and pulp necrosis, the expression of inflammatory cells has been linked to the upregulation of genes associated with macrophage synthesis and dendritic cell markers, particularly when related to interleukin levels [7].

The significant correlation between interleukin concentration and macrophage synthesis suggests a potential link to bone resorption observed in pulp necrosis. This relationship could provide valuable insights for early diagnosis and the development of targeted treatment strategies for this condition.

## Considerations

Given the current research on the subject, the pursuit of biomarkers appears to be a promising trend. In this context, the application of TNF-α as a potential biomarker for pulp necrosis shows promise. However, it is essential to further investigate whether a reliable correlation exists between interleukin concentration and the pathology to establish its clinical reliability. The interaction between systemic conditions, such as type 2 diabetes, and localized inflammatory responses significantly contributes to the complexity of pulp necrosis progression and diagnosis. Exploring the potential of TNF-α as a biomarker in these scenarios may facilitate early detection and optimize therapeutic strategies, particularly for individuals with comorbidities. Ultimately, studies focused on exploring TNF-α as a biomarker for pulp necrosis are crucial to clarify these points and validate its potential in clinical practice.
